# Unbalanced Growth, the DNA Replication Cycle and Discovery of Repair Replication

**DOI:** 10.3390/life13041052

**Published:** 2023-04-20

**Authors:** Philip C. Hanawalt

**Affiliations:** Department of Biology, Stanford University, Stanford, CA 94305, USA; hanawalt@stanford.edu

**Keywords:** bacterial cell cycle, DNA replication cycle, unbalanced growth, thymineless death, DNA repair replication, excision-repair

## Abstract

This article recounts my graduate research at Yale University (1954–1958) on unbalanced growth in *Eschericia coli* during thymine deprivation or following ultraviolet (UV) irradiation, with early evidence for the repair of UV-induced DNA damage. Follow-up studies in Copenhagen (1958–1960) in the laboratory of Ole Maaløe led to my discovery that the DNA replication cycle can be synchronized by inhibiting protein and RNA syntheses and that an RNA synthesis step is essential for initiation of the cycle, but not for its completion. This work set the stage for my subsequent research at Stanford University, where the repair replication of damaged DNA was documented, to provide compelling evidence for an excision-repair pathway. That universal pathway validates the requirement for the redundant information in the complementary strands of duplex DNA to ensure genomic stability.

## 1. Graduate Studies at Yale

The ground-breaking Watson/Crick model for the base-paired double-helical structure of DNA emerged in 1953–54, just as I completed an undergraduate physics major at Oberlin College and joined the Biophysics Department at Yale University for graduate study. I initiated my primary research project in the laboratory of Richard Setlow, a spectroscopist who was studying the effects of ultraviolet light (UV) on enzyme activities and on the inhibition of bacterial cell division. I was interested in focusing upon the effects of UV on growth and macromolecular synthesis in bacteria, and the molecular mechanisms for these effects. My eventual PhD thesis was titled “Macromolecular synthesis in *Escherichia coli* during conditions of unbalanced growth” [1].

Iverson and Giese had used colorimetric assays, (Indole for DNA and Orcinal for RNA), to follow the synthesis of DNA and RNA over an 8 h period after UV irradiation of *E. coli* [2]. To analyze in detail the early impact of UV upon DNA synthesis, I wanted to label the nascent DNA with radioactive thymine and use a thymine-deficient mutant to achieve maximum labeling efficiency with exogenous thymine. Seymour Cohen had studied a mutant strain, *E. coli* 15T-, deficient in thymidylate synthase, and had observed an exponential loss of viability with time in the absence of thymine. He named this phenomenon thymineless death (TLD) and attributed it to unbalanced growth, since protein synthesis and other metabolic activities continued unabated for a short period in the absence of DNA synthesis [3]. Balanced growth was defined by Allan Campbell as “over a time interval, if during that interval, every extensive property of the growing system increases by the same factor” [4]. Cohen provided the thymine-deficient mutant for my studies, and I used ^14^C-thymine to follow DNA synthesis. To follow other macromolecular syntheses for comparisons at the same time, I developed a sensitive microassay to resolve the incorporation of ^32^P, from ^32^P0_4,_ into DNA, RNA, and phospholipids [1,5]. The procedure, fully detailed in my thesis and publication, employs the precipitation of trichloroacetic acid-treated cells on collodion membrane filters, ethanol to remove phospholipids, and quantitative hydrolysis of RNA to mononucleotides by KOH, without loss of DNA The procedure was validated by comparison with colorimetric determinations.

I compared unbalanced growth during thymine deficiency to that following UV irradiation. I confirmed that UV inhibited DNA synthesis in *E. coli* and that it resumed after low UV doses, with a lag that increased as a function of the dose, while the synthesis of RNA and protein was less affected [1]. This was evidence that DNA synthesis could recover from the damage inflicted by UV, but we did not know what kind of damage was produced. Albert Kelner had discovered a phenomenon, termed photoreactivation, in which bacterial survival was greater if UV irradiation was followed by exposure to visible light [6]. I found that visible light exposure following UV shortened the lag in recovery of DNA synthesis, and I suggested that “the visible light facilitates repair of DNA integrity” [1,7]. That was my first use of the term, repair, but again, at that time we did not know what damage was being repaired, or even if the cellular lethality was primarily due to damage in DNA. I used monochromatic UV at 265 nm for the dose/response studies reported in the publication of my thesis work [1,8].

## 2. Postdoctoral Research at the University of Copenhagen

Upon completion of my graduate thesis, I was interested in a postdoctoral period to study synchronous growth in bacterial cultures so that I could look more closely at the time course of macromolecular syntheses during the cell cycle. (I also wanted the experience of living for a few years in another country!) Conveniently, it turned out that the acknowledged expert in synchronizing bacterial growth was Ole Maaløe, then at the State Serum Institute in Copenhagen. I applied and was accepted with an NIH postdoctoral fellowship. Just before I departed for Denmark, Seymour Cohen generously provided a further mutated strain of *E. coli* 15T-, not only deficient in the synthesis of thymine but also arginine and uracil. Thus, it was possible that I might compare the effects of RNA and protein syntheses on the phenomenon of TLD in the triple mutant strain.

Upon my arrival in Denmark, Ole Maaløe had just been appointed Professor and Director of the Institute of Microbiology at the University of Copenhagen, in a magnificent new building adjacent to the botanical garden overlooking Rosenberg Castle. He did not offer an immediate suggestion for my project, so I simply continued my studies on TLD, using the new triple mutant, which allowed me to look at the effects of protein and RNA synthesis on that process. Very few cells survived in the absence of thymine while protein and RNA synthesis continued for a limited period. However, when arginine and uracil were also deficient, TLD leveled off at about 3% survival. In the presence of thymine during the absence of protein and RNA synthesis, there was roughly a 40% increase in DNA followed by a plateau. If DNA synthesis was allowed for different periods, followed by thymine starvation, during the continued absence of protein synthesis, the survival level was increased until all the cells survived. This led to our hypothesis that the DNA replication cycle could be completed in the absence of protein and RNA synthesis and that thymine starvation only killed the cells that were actively carrying out DNA synthesis. The initiation of the cycle evidently required protein and RNA synthesis.

I needed to learn single-cell autoradiography for further analysis, so I visited my Yale graduate colleague, Robert van Tubergen. Bob was an expert in this technique and was currently in a postdoctoral position with Roy Markham in the UK. We carried out several preliminary experiments for me to master the approach and then I returned to Copenhagen to document the fraction of the population of individual cells that were synthesizing DNA in the absence of protein and RNA synthesis. This approach confirmed our model in which the number of cells actively carrying out DNA synthesis decreased with time under conditions in which protein and RNA synthesis were inhibited.

A new postdoc, Don Cummings, arrived in 1959 from Lloyd Kozloff’s laboratory at the University of Chicago to join Maaløe’s group, bringing experience in ultracentrifugation and the analysis of DNA replication by density labeling, as had been employed by Meselson and Stahl to prove the semiconservative mode of chromosomal DNA synthesis [9]. This approach validated our expectation that normal semiconservative DNA synthesis continued to completion in the absence of arginine and uracil, but that no new cycles of DNA replication were initiated under these conditions. The density labeling established that none of the initially labeled DNA molecules had begun a second cycle of replication. Thus, we had succeeded in synchronizing the bacterial DNA replication cycle.

Professor Maaløe presented the results from our studies in an invited lecture on “Control of normal DNA replication in bacteria” at the 1960 Cold Spring Harbor Symposium on “Cellular Regulatory Mechanisms” [10]. Unfortunately, the meeting was over-subscribed and I learned about it too late to participate. Several papers on my postdoctoral work were published later [11,12], and one of those papers [11] was selected as a Citation Classic by *Current Contents*, having been cited in over 530 publications between 1961 and 1964.

## 3. Second Postdoc, at the California Institute of Technology

I was interested in pursuing the density-labeling approach to study further details of DNA replication, and I received a fellowship from the American Cancer Society for a year with Robert Sinsheimer at Caltech, where I also became acquainted with Max Delbrook and Matt Meselson. We discussed the relationships of DNA molecules to the structure of the bacterial chromosome, and we postulated that the chromosome may consist of short segments of DNA, of about 7 million daltons, connected to each other by protein linkers. (It turned out that this was simply the characteristic size of DNA molecules obtained inadvertently upon shearing larger molecules during the loading of the ultracentrifuge rotor cell with a small-bore needle syringe.) John Cairns then showed by autoradiography in 1963 that the chromosome in *E. coli* consists of a single closed-circular duplex DNA molecule [13].

During my year at Caltech, Delbruck taught an exciting course on photobiology, in which he highlighted the recent discovery by Beukers and Berends, that UV irradiation causes co-valent dimerization of thymine molecules [14]; and it was soon shown that cyclobutane pyrimidine dimers could be formed between adjacent pyrimidines in DNA. So, there was finally a documented candidate for a responsible DNA lesion that caused growth inhibition and lethality in UV-irradiated bacterial cells. In 1962, Wulff and Rupert reported that photoreactivation involves the direct reversal of pyrimidine dimers to free thymine in situ, without affecting the phosphodiester backbone of DNA [15]. That finding supported my earlier speculation that photoreactivating light facilitated the repair of damage that was hindering the resumption of DNA synthesis in the UV-irradiated cells. I obtained further experience in density-labeling technology, with mentorship from Sinsheimer and Jerry Vinograd, while also searching unsuccessfully for physical changes in DNA during thymine deprivation.

## 4. Faculty Appointment at Stanford University

In September 1961, I joined the Biophysics Laboratory at Stanford University as a Research Biophysicist and Lecturer. I initiated a new graduate course in Molecular Biophysics and began undergraduate teaching in Arthur Giese’s Cell Physiology course, remembering that my graduate research had followed upon his early studies! In 1965, I was promoted to Associate Professor with tenure in the Department of Biological Sciences.

Two incoming biophysics graduate students, Dan Ray and David Pettijohn, joined me in 1962 to compare DNA replication under normal growth conditions with that following UV irradiation. Dan and I were able to isolate partially replicated growing fork DNA fragments in *E. coli* through ^32^P pulse labeling of DNA during bacterial growth in a medium containing 5-bromouracil, replacing thymine to density-label replicating DNA, and analysis by density-gradient equilibrium sedimentation. We found that the fork-containing DNA fragments were selectively sensitive to shearing into replicated and unreplicated sub-fragments [16].

David and I focused on the qualitative nature of DNA replication in UV-irradiated bacteria. We had expected to isolate DNA segments in which the replication fork was blocked by pyrimidine dimers, and that these would appear as partially replicated fragments during density labeling. We confirmed that semiconservative DNA synthesis was inhibited, but surprisingly, a new mode of non-conservative DNA synthesis appeared, in which much of the density-labeled nascent DNA was in very short segments, too short to significantly shift the density of DNA fragments containing them. Intentional shearing did not resolve them into fragments of different densities, leading to our conclusion that the nascent DNA was in short “patches” embedded in the parental DNA fragments [17].

Meanwhile, Richard Setlow had been continuing the studies on the inhibition of DNA synthesis that I had initiated at Yale; but now, with the knowledge that UV exposure induced pyrimidine dimers and with the availability of a UV-sensitive mutant bacterial strain, *E. coli* B_S-1_, isolated by Ruth Hill [18], Setlow identified very short oligomers containing pyrimidine dimers that were excised from the DNA in the UV-irradiated parental bacterial strain, but not in the UV-sensitive mutant. He proposed an excision-repair mechanism to explain his results [19]. Paul Howard-Flanders obtained the same results using wild-type and UV-sensitive mutants of *E. coli* K12 strains [20]. Of course, the excision of those damaged DNA oligomers would leave another lesion, a gap in the parental DNA strand.

David Pettijohn and I had discovered the second step in excision-repair, the filling of those gaps by repair replication, a non-conservative mode, using the undamaged parental strand as a template [21]. Unlike the control of chromosomal DNA replication, repair replication was not affected by the cell cycle. It was just as efficient in cells that had completed the cycle as in those undergoing chromosomal DNA synthesis [22]. We concluded that the Watson/Crick duplex DNA structure was needed, not only for sequential replication of the genome but also that it was absolutely essential for the repair of damage in the respective DNA strands. My colleague, Robert Haynes, and I provided a short history of the emerging field of DNA repair in *Scientific American* [23]. The studies in my laboratory on repair replication were then presented in the 1968 Cold Spring Harbor Symposium [24], and I published a broader review on cellular responses to photochemical damage in a chapter in a book on Photophysiology, edited by Arthur Giese [25].

## 5. Reflections on the Primordial Genome and the Need for DNA Repair

DNA is not unusually stable, as had been originally assumed, and we now know that multiple repair pathways are needed to maintain the integrity of the genome. The genome is subject to endogenous oxidative damage, and DNA is also intrinsically unstable due to spontaneous depurination, which generates a basic site that must be repaired. In contrast, the depurination of RNA is very much slower, while RNA is more prone than DNA to strand breaks and degradation (see the classic review by Tomas Lindahl [26]). For the existence of living cells, there must have been informational nucleic acids of sufficient lengths to encode the needed proteins/enzymes and ribozymes. In abiogenic synthesis experiments, only relatively short DNA molecules have been generated. Perhaps longer informational nucleic acid chains were formed by the ligation of short ones. An undergraduate physics student, Roger Lewis, and I carried out a proof-of-principle study in which we demonstrated that UV irradiation of duplex DNA, in which short thymine-containing oligomers were paired with a long adenine-containing strand, could result in the thymine–dimer linkage of the oligomers, to leave a strand break at each linkage site without any loss of continuity in the other strand [27].

Nucleic acid repair would have been essential for the origin of life as well as for its persistence, and the redundant double-strand genome must have been required at the very beginning of life. (This of course assumes that the original informational genome consisted of nucleic acids!) I think it likely that the first functional genomes were composed of a mixture of ribonucleotides and deoxyribonucleotides, since both were present in the primordial “stew”, and that this combination may have helped to ensure both the informational and the structural stability of the first genomes. The primordial earth was bombarded by a high flux of UV light, not attenuated by an ozone layer. It is likely that sunlight photochemistry played an important role in the origin of life, while paradoxically, it was also one of the principal threats to its persistence [28].
